# The Apoplast: A Key Player in Plant Survival

**DOI:** 10.3390/antiox9070604

**Published:** 2020-07-10

**Authors:** Atefeh Farvardin, Ana Isabel González-Hernández, Eugenio Llorens, Pilar García-Agustín, Loredana Scalschi, Begonya Vicedo

**Affiliations:** Grupo de Bioquímica y Biotecnología, Departamento de Ciencias Agrarias y del Medio Natural, Universitat Jaume I de Castellón, Avenida de Vicent Sos Baynat, s/n, 12071 Castellón de la Plana, Spain; farvardi@uji.es (A.F.); gonzalan@uji.es (A.I.G.-H.); ellorens@uji.es (E.L.); garciap@uji.es (P.G.-A.)

**Keywords:** apoplast, plant defense, ROS, antioxidants, proteins, peptides, hormones, MAMPs

## Abstract

The apoplast comprises the intercellular space, the cell walls, and the xylem. Important functions for the plant, such as nutrient and water transport, cellulose synthesis, and the synthesis of molecules involved in plant defense against both biotic and abiotic stresses, take place in it. The most important molecules are ROS, antioxidants, proteins, and hormones. Even though only a small quantity of ROS is localized within the apoplast, apoplastic ROS have an important role in plant development and plant responses to various stress conditions. In the apoplast, like in the intracellular cell compartments, a specific set of antioxidants can be found that can detoxify the different types of ROS produced in it. These scavenging ROS components confer stress tolerance and avoid cellular damage. Moreover, the production and accumulation of proteins and peptides in the apoplast take place in response to various stresses. Hormones are also present in the apoplast where they perform important functions. In addition, the apoplast is also the space where microbe-associated molecular Patterns (MAMPs) are secreted by pathogens. In summary, the diversity of molecules found in the apoplast highlights its importance in the survival of plant cells.

## 1. Introduction

**Figure 1:**
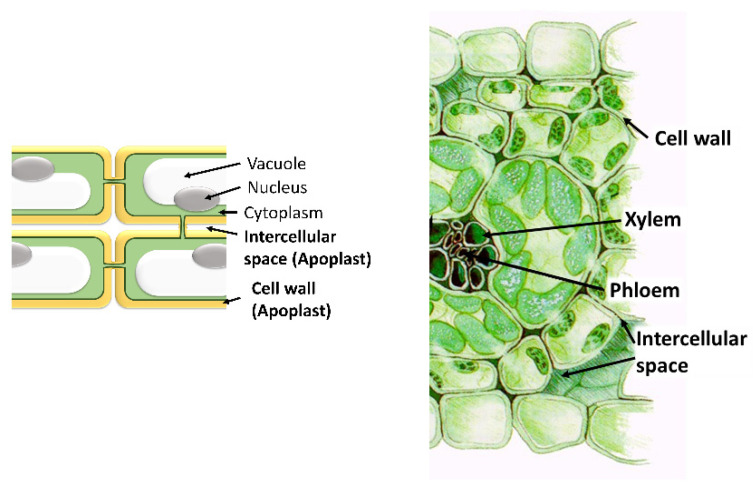
Spaces and structures that form the apoplast in plants. Adapted from: Mluisalozanopulido (CC BY-SA 3.0 (https://creativecommons.org/licenses/by-sa/3.0)).

**Figure 2:**
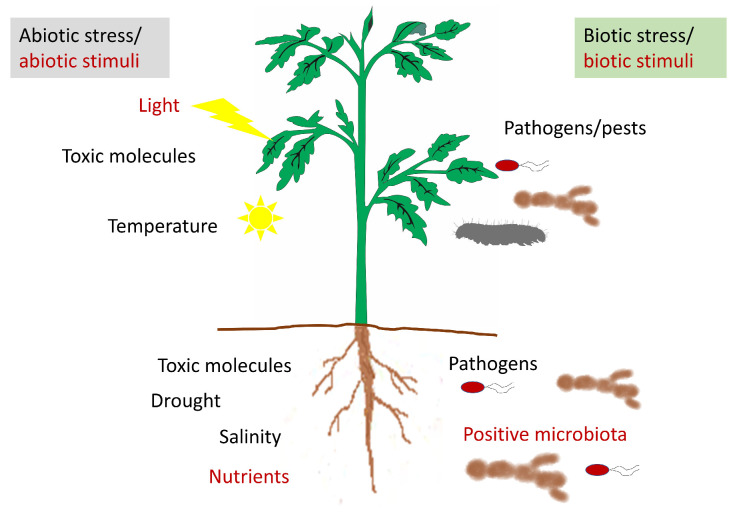
Representative diagram of the stresses and stimuli perceived by the plant that could modify the apoplastic content.

The term apoplast was coined by the German scientist E. Münch in 1930 [1]. He considered the apoplast as the intercellular space involved only in water and nutrient transport and separated the “dead” apoplast from the “living” symplast. Nowadays, the apoplast is defined as the intercellular space filled with gas and water, contained between cell membranes, the interfibrillar and intermicellar space of the cell walls, and the xylem extending to the rhizoplane and cuticle of the outer plant surface (Figure 1). The apoplast is involved in a vast number of functions, since its dynamic nature allows for many reactions of great importance for the plant to take place in it. Among these functions, we can mention nutrient and water transport and the syntheses of components of the cell wall and other molecules. Among them, the synthesis of molecules involved in plant defense against both biotic (phytoalexins, PR, proteins, enzymes) and abiotic stresses are important, since it is in the apoplast where the environmental changes are detected (Figure 2).

The synthesis of the cell wall takes place in the apoplast, after the liberation of the cell wall components through the plasma membrane. This produces the modification, degradation, and reorganization of cell wall polymers, depending on the state of cell development and differentiation. The main components of the cell wall are pectin, cellulose, and hemicellulose, but other components such as xyloglucan or phenolic compounds can be part of it. It is known that some of the steps involved in wall assembly are enzymatic, although there are other steps that seem to simply occur by chemical changes produced in the structure of its components that allow their interaction or deposition [2,3,4]. The content of the cell wall is constantly changing and can be modified according to the stimuli that the plant cell is receiving through the apoplast. That way, both growth activation and the direction of growth or its arrest are regulated by the presence of certain molecules in this intercellular space [5]. In addition to the general constituents of the cell wall, when the plant faces adverse stimuli such as different pathogens, the cell wall can be reinforced with the deposition of callose.

Since the apoplast represents the first barrier between the cell and the surrounding environment, its role in cell nutrition is also of great importance. It has been shown that the apoplast is involved in the transport of glucose produced during photosynthesis and of amino acids to the phloem [6,7]. In the same way, Sattelmacher [8] reviewed the role of the apoplast in plant mineral nutrition, analyzing its role in the acquisition of nutrients in root cells, and important aspects such as Na^+^ toxicity and Al^3+^ tolerance. Moreover, he also defined its role in long-distance transport and maintenance of the microbiota through the xylem, and short-distance storage of nutrients in the leaves. The apoplastic content of the leaves is very rich in different molecules and may vary according to the state of the plant, which points to its fundamental role in plant response to external stimuli such as the presence of pathogens [9,10,11].

Hoson [12] attributed an important role to the apoplast in the communication of plants with the external environment, not only at the level of response but also at the level of stimuli perception and their transduction, enabling, that way, plant adaptation to stress. One of the abiotic stresses to which the plant may be subjected is the presence of toxic molecules in the environment. Related to this, it was observed that the apoplastic content plays an important role in overcoming aluminum toxicity, especially through modification of its components avoiding aluminum binding to the wall [13,14]. That way, the changes in the composition prevent the inhibition of root elongation and confer resistance against this stress. It was also shown that other abiotic stresses such as drought or salinity can induce changes in the apoplastic content that could help the plant to overcome them. Moreover, Geilfus [15] suggested that the alkalinization of the pH of the apoplast after exposure to these stresses could play an important role since the transient increase in the pH of the apoplast reduces the stomatal aperture or even may inhibit stomatal opening via effects related to abscisic acid (ABA).

The apoplast also serves as a niche for microbes [7,16]. On the one hand, the endophytic organisms, which tend to be present at low levels, are beneficial for the plant. It has been demonstrated recently by different authors that this symbiosis with endophytic microorganisms play an important role in growth and production, and can help the plant to overcome different stresses [17,18,19].

On the other hand, the content of the apoplastic fluid is rich in nutrients that allow for populations of pathogenic microorganisms to develop in it [9]. However, it has been shown that changes in nutrient composition and metabolism within the apoplast itself may favor pathogen control [11,20]. The apoplastic defense is related to the set of molecules that are either present in the apoplast or secreted in the presence of the pathogen that contributes to plant defense against it [21]. These include reactive oxygen species, toxic compounds, and anti-pathogenic protein molecules such as “pathogenesis related” (PR), other proteins, peptides, and small molecules that are secreted into the apoplastic space. In the plant defense system, through the ETI (effectors triggered immunity) plants are able to recognize the effector molecules secreted by the pathogen through immunoreceptors that are nucleotide-binding leucine-rich repeat proteins (NLR), the synthesis of which leads to a strong apoplastic defense. The interaction between apoplastic defense and control in stomatal closure has been studied, showing that NLR function is cell autonomous in guard cells for stomatal closure defense and non-cell autonomous for apoplastic defense and in the latter case it could affect the defense of the entire plant [22].

This review was written to gather an overview of the recent advances in the field and to gain novel insights into key mechanisms and components, such as ROS, antioxidants, proteins/peptides, and hormones that determine apoplastic immunity and modulate plant–pathogen interactions.

## 2. Apoplastic ROS Production and Their Role to Overcome Stress

Reactive oxygen species (ROS) are natural products generated in the cells by many metabolic reactions [23]. When first discovered, they were considered deleterious to the cell biomolecules. Nowadays, it is known that ROS play an important role in plant signaling, controlling not only vital processes such as plant growth and development but also plant response to both biotic and abiotic stresses [24,25,26,27].

ROS includes singlet oxygen (^1^O_2_), superoxide ion (O_2_^•−^), hydrogen peroxide (H_2_O_2_), and hydroxyl radical (^•^OH), and other organic molecules, such as organic hydroperoxides (ROOH) [28]. However, H_2_O_2_ is the most stable molecule and acts as a signal to trigger several cellular responses, such as the modulation of root development or the response against different stresses [29,30].

ROS are reported to be produced in different subcellular compartments, such as the plasma membrane, mitochondria, chloroplasts, and peroxisomes. Different studies also mention the apoplast as a site for ROS generation, although at much lower levels than in the cell [31,32]. The apoplastic ROS (apROS) is produced by several groups of enzymes, such as amine oxidases, lipoxygenases, and oxalate oxidases, but the most recognized enzymes are the cell wall peroxidases and the plasma membrane NADPH oxidases [33].

Apoplastic **peroxidases** **(also known as POXs, Prx, POD, Px or PER)** are a class of enzymes that belong to a large class III peroxidases, responsible for the formation and degradation of ROS induced by stress [34]. They are heme oxidoreductases that catalyze O_2_ conversion to O_2_^•−^ or H_2_O_2_ using apoplastic reductants or ^•^OH production from H_2_O_2_ and O_2_^•−^ [33]. They play an important role in a broad range of developmental and physiological processes, such as cell elongation, auxin metabolism, lignin and suberin formation, cross-linking of cell wall components, and synthesis of phytoalexins; and they participate in ROS and RNS (reactive nitrogen species) metabolism [35]. Moreover, an increase in the activity of class III peroxidases was observed after exposure to various environmental stresses, such as salt, wounding, temperature, phosphate starvation, or potassium deficiency [36,37,38]. For example, *Arabidopsis thaliana* overexpressing the peroxidase *Prx3* showed increased tolerance to salt. In the same way, the expression of *CrPrx1* and *CrPrx* from *Catharanthus roseus* in *Nicotiana tabacum* lead to enhanced tolerance to cold stress treatment. In addition, POXs are a well-known class of PR proteins, being induced in host plant tissues by pathogen infection. They belong to the PR-protein 9 subfamily and help to limit the spreading of the infection through the formation of physical barriers or by counterattacking with a large production of ROS. POXs can create physical barriers to restrict pathogen invasion in host tissues by catalyzing the cross-linking of cell wall components which finally leads to cell wall rigidification [39]. This process also occurs in response to wounding or to environmental constraints or simply as a part of the normal cell wall development during growth, differentiation, and senescence [40,41]. Therefore, *POX* expression results in plant defense either by building up stronger walls or by production of ROS against different stress factors. Related to this, it was observed that knockdown of *Prx33* and *Prx34* genes in *A. thaliana* caused increased susceptibility to both fungal and bacterial pathogens, with an impaired oxidative burst, while expression of *CaPO2* (*CaPrx2*) from *Capsicum annuum* in *A. thaliana* was found to enhance disease resistance. Furthermore, ROS produced by class III POXs was reported to play an important role in *A. thaliana* PAMP-triggered immunity (PTI), while the overexpression of these peroxidases provides resistance against *Botrytis cinerea* infection [37]. In the same way, a positive correlation between peroxidase activity and resistance to *Pseudomonas syringae* pv. *tabaci* disease was observed in tobacco plants [38].

Plant **NADPH oxidases, named respiratory burst oxidase homologs (RBOHs)**, are located in the plasma membrane and catalyze the production of apoplastic O_2_^•−^ by transferring electrons from cytosolic NADPH or NADH to apoplastic O_2_ [42]. The produced O_2_^•−^ can further be converted to H_2_O_2_ by superoxide dismutase (SOD) [42]. NADPH oxidases have been implicated in abiotic and biotic stress responses, and in the development in different plant species. These enzymes have been studied in detail in *Arabidopsis thaliana* where ten isoforms have been identified [43]. Among them, RBOHD and RBOHF seem to play crucial roles in the generation of ROS in response to pathogen attack and abiotic stress, while RBOHC, RBOHH and RBOHJ are more related to development. Deficient-mutants in RBOHs such as rbohD and rbohF have been a valuable tool in the study of ROS-abiotic stress interactions [44]. The activation of RBOH enzymes such as RBOHD requires an increase of intracellular calcium, and it can be regulated by ABA, which has been previously described; that regulates ROS production through the RBOH enzymes (RBOHD and RBOHF) [28]. As mentioned above, these enzymes are involved in ROS production in response to biotic and abiotic stresses and they are required for initiation and rapid propagation as systemic signaling between cells, while being dependent on H_2_O_2_ accumulation in the apoplast to produce a “ROS wave” [45,46,47,48]. This wave can prime neighboring cells in joint action with other molecules such as hormones, mediating, that way, plant acclimation to abiotic stresses [45]. For example, [49] demonstrated that, in tomato plants, this acclimation-induced cross-tolerance process was related to an increase in H_2_O_2_ production dependent on *RBOH1* at the apoplast and the subsequent activation of the mitogen-activated protein kinases. That way, NH_4_^+^-fed tomato plants displayed basal stomatal closure produced by H_2_O_2_ from enhanced *CuAO* and *RBOH1* gene expression, which contributes to protecting the plant against *Pseudomonas syringae*, reducing disease symptoms and inducing the oxidative burst upon the infection [50]. Moreover, this NH_4_^+^-induced oxidative burst seems to be alleviated by the below-mentioned antioxidant enzyme Mn-SOD activity [51].

**Oxalate oxidases** belong to the germin-like protein family (GLP) and catalyze the oxidation of oxalate to H_2_O_2_ and CO_2_. Plants with higher oxalate oxidase activity were found to be more resistant to plant pathogens, such as the enhanced resistance to *Rhizoctonia solani* with an overexpressed *oxalate oxidase 4* (*Osoxo4*) gene in rice plants [52,53]. Moreover, oxalate oxidase-mediated H_2_O_2_ production in root cells was shown to be important for drought stress acclimation [44].

Apoplastic **amine oxidases (AOs)** catalyze the oxidative deamination of the essential compounds polyamines (PAs), producing aldehydes, ammonia, and H_2_O_2_. They include the copper-containing amine oxidases (CuAOs) and the flavin-containing PA oxidases (PAOs). Both enzymes have been shown to play a key role as a source of H_2_O_2_ during plant development and differentiation and in defense mechanisms against pathogens, abiotic stress, and symbiotic interactions [54,55]. The stress signaling can lead to an enhancement in PA levels inside the cell that are released into the apoplast and oxidized producing H_2_O_2_. If this H_2_O_2_ concentration is high, the programmed cell death (PCD) event occurs, while a slight H_2_O_2_ production induces ROS-scavenging enzymes’ action [56]. For example, in chickpeas, the resistance against the fungus *Ascochyta rabiei* was related to higher expression of *CuAO* compared with susceptible cultivars [57]. However, both CuAO and PAO could act as PA back-converters in peroxisomes [58].

Other sources of apoplastic O_2_ are the lipoxygenases (LOX), which are nonheme iron-containing dioxygenases [59]. LOX activity can produce lipid peroxidation, leading to the formation of polyunsaturated fatty acids (PUFA) which can generate ROS [33]. It was observed that LOX activity and lipid peroxidation was increased in maize leaves during low temperatures, suggesting that lipoxygenase-mediated peroxidation of membrane lipids contributes to the oxidative damage occurring in chill-stressed maize leaves [60]. Moreover, apoplastic lipoxygenases were shown to be involved in the oxidative burst together with peroxidases and amine oxidases in *Pisum sativum* seedlings upon wounding [61]. Besides, an elevated LOX activity was observed during infection of pea roots with the cyst nematode *Heterodera goettingiana* [62].

Even though apoplastic ROS (apROS) represents only a minor part of the total ROS level of cells, it has great importance since it regulates whole-plant growth by controlling cell division rate and cell elongation [33]. Moreover, an important function of apROS is the regulation of stomatal movement, and cell wall reinforcement by inducing lignin biosynthesis [28,63]. In the same way, apROS are involved in signal transduction from extracellular spaces to the cell interior and may directly eliminate invading pathogens [33]. This is achieved through the activation of immune receptors localized in the plasma membrane such as the receptor-like kinases (RLKs). It was observed that apROS production often occurs following activation of RLK signaling in different cellular processes [64]. Moreover, these ROS can further enter inside the plant cell as H_2_O_2_ through membrane aquaporin channels [64]. ApROS were also found to regulate callose deposition at the cell wall, increasing, that way, plant resistance to fungal pathogens. In addition, ROS’ ability to modify the structures of several proteins which are known to play important roles in plant immunity has also been reported [28].

## 3. Antioxidants in the Apoplast

As mentioned above, ROS can be at the same time beneficial and deleterious for the plant, since they can act as secondary messengers in different physiological processes [65]. However, they can also induce oxidative damage under several environmental stress conditions, such as salinity, drought, cold, heavy metals, and UV irradiation. In the latter case, ROS accumulation may cause many cellular damages that consist of degradation of several biomolecules such as pigments, proteins, lipids, carbohydrates, and DNA, which finally leads to PCD. It was revealed that a high concentration of ROS has a toxic role in plant cells, so the actions of ROS scavengers and antioxidant enzymes are required to avoid its toxicity [65]. The components of the antioxidant machinery can be broadly divided into enzymatic and non-enzymatic. The relevant ROS-scavenging enzymes are superoxide dismutase (SOD), catalase (CAT), ascorbate peroxidase (APX), monodehydroascorbate reductase (MDHAR), dehydroascorbate reductase (DHAR), and glutathione reductase (GR) (Table 1). Moreover, the non-enzymatic antioxidants such as ascorbate, glutathione, proline, phenolic compounds, and polyamines are also required to avoid cell toxicity [66] (Table 2). **Superoxide dismutases (SOD)** are enzymes that convert O_2_^•−^ into H_2_O_2_, which is less harmful to the plant. Therefore, they are considered as the first line of detoxification of ROS. SODs are classified according to the metal cofactor used by the enzyme, as manganese (Mn-SOD), iron (Fe-SOD), and copper–zinc (Cu/Zn-SOD) dependent, although Cu/ZnSOD was identified as the most active apoplastic isoform. Furthermore, many studies have reported that these enzymes confer resistance to abiotic stress. These studies highlight the fact that the tolerance level of plants is positively correlated with SOD activity and with the number of SOD isoforms. SOD is up-regulated in many plant species by various types of abiotic stress-inducing factors, such as drought, salt, and heavy metals, in a large number of crops, such as, tomato, wheat, barley, and citrus [67]. Related to this, it was shown that apROS production in wheat root decreased upon a copper treatment, which was correlated with an increase in SOD activity [68]. Moreover, Garcia et al. [69] found a positive correlation between SOD activity and salt-tolerance when studying the response of the apoplastic antioxidant systems in root and leaf tissues from a salt-sensitive and a salt-resistant onion genotype in response to salinity. Furthermore, increased SOD activity was also observed in response to biotic stress. Vanacker et al. [70] showed that the activities of several antioxidant enzymes such as SOD, CAT, APX, DHAR, MDHAR, and GR were induced in the apoplasts of barley and oat leaves 24 h after inoculation with the biotrophic fungal pathogen *Blumeria graminis* [71]. Furthermore, *Trichoderma harzianum* inoculated sunflower plants were more resistant to *Rhizoctonia solani*. This resistance was accompanied by an increase in SOD activity in root apoplast seven days post-inoculation [72].

**Catalases (CAT)** are the only and the main scavenging enzymes that do not require any reductant for ROS decomposition and directly decompose H_2_O_2_ to water and O_2_ [33]. Moreover, it seems that there is a link between CAT activity and ROS production by RBOH and POXsin the apoplast [87]. In addition, CAT seems to have the highest enzyme activity in this intercellular space, reaching around 0.2% to 2% of the total [70,71,88]. Several studies have shown that an increase of CAT is essential for plant defense against different stresses, since its correct functioning could avoid the damages produced by ROS accumulation. For example, the overexpression of a catalase gene isolated from *Cucumis sativum* (*CsCAT3*) in *E. coli* could increase the tolerance to cold, heat, osmotic, or salinity stresses [89]. Moreover, it was also suggested that SA binds this enzyme, reducing its activity such as it occurs in NO_3_^−^ treated citrus plants exposed to salinity compared to NH_4_^+^ treated plants that showed an increase in CAT activity, allowing plants to tolerate better the salinity-induced oxidative damage [90].

Within the antioxidant system in plants, the ascorbate–glutathione (Asc-GSH) pathway plays a major role in regulating ROS. Four enzymes are engaged in this pathway: **ascorbate peroxidase (APX), dehydroascorbate reductase (DHAR), monodehydroascorbate reductase (MDHAR), and glutathione reductase (GR)**. These enzymes catalyze the reduction of H_2_O_2_ to water using ascorbate (Asc) and glutathione (GSH) as electron donors. In the Asc-GSH pathway the reduction of H_2_O_2_ is ultimately linked to NAD(P)H oxidation. All the enzymes of the Asc-GSH pathway were found in the apoplasts of barley, oat, and pea leaves, but their activity was much lower when compared to their symplastic activity [70,71]. However, in the apoplasts of other plants, not all Asc-GSH pathway enzymes were found to be active; therefore, only certain enzymatic reactions may take place [91].

**Ascorbate peroxidase (APX)** is a key enzyme of the Asc-GSH pathway [92]. APX catalyzes the reduction of H_2_O_2_ to water using Asc as an electron donor. Based on amino acid composition, five isoforms of APX have been identified in plants: cytosolic (cAPX), mitochondrial (mitAPX), chloroplastic (chlAPX: stromal-APX and thylakoidal-APX), and peroxisomal/glyoxysomal (mAPX) [93]. In Arabidopsis only the cytosolic isoform was found to be secreted to the apoplast [94]. It was observed that the expression of APX encoding genes takes place under abiotic stresses (salt, drought, heat, cold, UV radiations, oxidative stress, etc.) and under biotic stresses (pathogen attack, herbivory). The role of APX isoforms concerning different types of abiotic stresses is reviewed in detail by Pandey et al. [93]. An increase in APX in the leaf apoplast of *Ctenanthe setosa* in response to drought stress was observed [95]. Related to biotic stress, in the Plum pox virus-resistant apricot cultivar Stark Early Orange, a rise in class I APX and strong increases in POX and SOD activities were noticed in the apoplastic compartment [96].

**Monodehydroascorbate reductase (MDHAR)** is responsible for regenerating Asc from monodehydroascorbate (MDHA) using NADPH as a reducing agent. Therefore, MDHAR is an important enzyme responsible for maintaining the reduced Asc pool, by optimizing the recycling of oxidized Asc and is a key component in the stress tolerance profile of a plant. The activity of MDHAR is induced by various stress conditions [97,98,99]. Although MDHAR activity has been detected in several cell compartments, such as chloroplasts, mitochondria, peroxisomes, and the cytosol, little evidence exists about its presence in the apoplast. Related to this, Bindschedler et al. have shown that one of the six MDHAR isoforms found in Arabidopsis seems to be localized in the apoplastic space [76].

**Dehydroascorbate reductase (DHAR)** reduces dehydroascorbate (DHA) to Asc using reduced GSH as an electron donor [99]. Therefore, DHAR is involved together with MDHAR in regenerating the Asc pool, maintaining, that way, the Asc levels in both symplast and apoplast. Overexpression of DHAR has highlighted its important roles in ascorbate regeneration and responses to environmental stresses. DHAR functions in plant defense, growth, and development were reviewed in detail by Dingl et al. [78].

**Glutathione reductase (GR)** is also an important component of the antioxidant machinery. GR catalyzes the reduction of glutathione disulfide (GSSG) to GSH using NADPH as a reductant, which is of great importance for maintaining the cellular redox balance of GSH/GSSG. GR was found to play a positive role in tolerance to abiotic stress [79]. For example, an increase in GR in the leaf apoplast of *Ctenanthe setosa* in response to drought stress was described [95]. As mentioned above, Vanacker et al. [71] observed an increase in GR in barley and oat apoplast upon *Blumeria graminis* infection.

**Ascorbic acid (AsA)** or vitamin C is an antioxidant that plays an important role in plant growth and environmental stress tolerance. The physiologically active form of AsA is termed ascorbate (Asc). The most abundant pool of Asc is found in the cytoplasm, while in the apoplastic space of most plants, the Asc content was estimated to reach up to 10% of the total cellular pool. Despite this, the apoplastic fraction of AsA is considered of great importance for oxidative stress signaling. The antioxidant capacity of the apoplast is generally low and despite the presence of other antioxidants, it is highly dependent on AsA pools [100]. To protect against the deleterious effects of ROS, the AsA pools are required to be maintained in a reduced state. For this purpose, Asc is oxidized to MDHA by the action of APX and then to DHA by non-enzymatic dissociation. The oxidized forms of Asc are converted back to reduced Asc by MDHAR using NADH or NADPH as an electron donor and by DHAR using reduced GSH as an electron donor, respectively. Due to its apoplastic localization, AsA plays an important role in stress perception, redox homeostasis, and regulation of oxidative stress and plant responses under normal or abiotic stress conditions. AsA has been shown to be effective in improving stress tolerance in plants. Several examples can be found in Akram et al. [100].

Apart from the AsA, there are many non-enzymatic compounds that intercept and complete free radical reactions and possess the capacity to decrease substrates for antioxidant enzymes, reducing the deleterious effects of ROS in plants. Several examples of these antioxidants are **glutathione, proline, phenolic compounds, and polyamines**, among others. GSH is known as a powerful scavenger that has diverse functions, such as protecting cell membranes, preventing oxidative denaturation of proteins, and being a substrate for glutathione peroxidase and S-transferase [101]. Thus, GSH is also involved in stress responses, as was shown in *Arabidopsis thaliana* plants, where an increase of GSH was related to drought and salt tolerance [102]. Moreover, a relation of GSH with heat tolerance has been suggested, since an accumulation of GSH levels was also observed in wheat plants under heat stress [103], and a high GSH was observed in heat-tolerant wheat genotypes [104,105]. In addition, proline is a proteinogenic amino acid that possesses osmoprotective activity, and an antioxidant, a metal chelator, and a signaling molecule during stresses [106]. When this amino acid was exogenously supplied, a reduction of ROS was observed in the roots of *Arabidopsis thaliana* [107]. Thereby, proline application alleviates salinity stress in *Vicia faba* plants [108]. Moreover, an accumulation of proline was observed in drought-tolerant and salinity-tolerant chickpea and rice plants, respectively [109,110]. Finally, increased concentrations of proline in response to drought stress were detected in both the apoplastic and symplastic compartments of leaves from drought-tolerant and drought-sensitive cultivars of the common bean [82]. In the same way, an increase in the apoplastic proline content was observed by Saglam et al. [111] in *Ctenanthe setosa* plants submitted to drought stress. Other non-enzymatic compounds are phenolic compounds, secondary metabolites that donate electrons or atoms to ROS, and inhibiting oxidizing enzyme activities [112,113]. The accumulation of these compounds in different crops growing under heavy metals, salt, or drought stresses has been widely studied [114]. Finally, the role of polyamines should be pointed out, since they are small aliphatic amines involved in the antioxidant response [56], but as mentioned above, their catabolism also produces H_2_O_2_ by AOs action in the apoplast [58].

To sum up, it is important to mention that there is a relation between the different antioxidants mentioned, and the combined action of the whole set of antioxidant defense compounds produces an enhancement of the response to cope with environmental stresses.

## 4. Apoplastic Proteins and Peptides in Modulating Plant–Pathogen Interactions. Microbe-Associated Molecular Patterns (MAMPs) of Proteic Nature

The most abundant proteins related to defense are the pathogen-related proteins (PR-proteins) that represent 23–33% of the total apoplastic fluid proteins (AFPs) [115]. The production and accumulation of PR-proteins are produced by various stresses, including biotic/biological stresses and abiotic/non-biological stresses [116]. The plant PR-proteins are divided into 17 groups (Table 3). Moreover, based on the amino acid sequence and the biochemical activity identified during the last decade, they also include novel peptide families. The PR peptides include proteinase inhibitors (PR-6 family), plant defensins (PR-12 family), thionins (PR-13 family), and lipid transfer proteins (PR-14 family) [117,118].

Besides the plant PR-peptides mentioned above, the regulation of signaling pathways by small peptides is a central theme in molecular biology. The apoplast is also the space where the microbe-associated molecular patterns (MAMPs), by plant cell surface-localized pattern recognition receptors (PRRS), are initially determined [135].

### 4.1. Apoplastic Proteins Related to Plant Defense

Nowadays, PR-proteins, based on their protein sequences similarities and other biological features, show diverse functions, such as β-1,3-glucanase (PR-2), chitinases (PR-3), thaumatin-like (PR-5), and peroxidases (PR-9) [118,136].

Despite their differential fungicidal activity, PR-1 proteins are often used as markers for salicylic acid (SA)-mediated disease resistance [115,119]. PR-1 proteins have been identified in *Oryza sativa*, *Nicotiana tabacum*, and Arabidopsis, and often have a molecular weight of 14 to 17 kDa [137]. Although PR-1 proteins can have different properties and differ substantially in biological activity, all of them have a similar structure and are classified in the same family based on sequence homology [39].

Plant β-1,3-glucanases are grouped in the PR-2 family of PR proteins and play an imperative role in plant defense, and the usual processes of plant growth. These proteins have a size from 33 to 44 kDa, with both acidic and basic isoforms [120]. Based on the amino acid sequencing, structural features, and cellular localizations, the glucanases have been classified into three major classes and two minor classes [138,139,140]. Most of classes II, III, and IV of β-1,3-glucanases acidic proteins were found and secreted into the extracellular spaces. Moreover, several studies have shown that the synthesis of β-1,3-glucanases is stimulated by pathogen infections and it makes the plant resistant to fungal pathogens either alone or in association with other proteins, including chitinases, peroxidases, and thaumatin-like proteins [141,142,143]. Furthermore, it has been suggested that β-1,3-glucanases act as the key enzymes in the lysis of phytopathogenic fungal cell walls during the antagonistic action by hydrolyzing the O-glycosidic linkages of β-glucan chains [144,145]. Besides, the results shown by Floerl et al. [10] demonstrated PR-2 apoplastic activity in susceptible primary leaves of *Arabidopsis thaliana* 25 days after inoculation with *Verticillum longisporum*. Additionally, in the related article they reported that β-1, 3-glucanases are increased in wheat plants (*Triticum aestivum*) in response to *Zymoseptoria tritici* infection [122].

Chitinases are a huge and diverse group of enzymes that have been classified into two main categories—endochitinases and exochitinases, and based on their primary structures, plant chitinases have been divided into seven classes, class I through VII [146,147]. Plant chitinases which represent a major group of PR-proteins are expressed in response to environmental stresses and the attack of phytopathogens and secreted in extracellular space [148,149,150,151,152]. Moreover, several studies have shown that extracellular chitinases induce chitinase biosynthesis by detecting fungal elicitors and block the spreading hyphae invading intercellular spaces as well. For example, Petriccione, et al. [153] reported that extracellular chitinase was induced in infected *Actinidia deliciosa* leaves with *Pseudomonas syringae*. Furthermore, Pechanova et al. [154] showed that class IV chitinase and acidic class III chitinase were accumulated in leaves of poplar upon water stress. They demonstrated that three different type III chitinases were detected in leaf apoplasts, while the poplar stem apoplast contains only one single type III chitinase. In addition, apoplastic chitinases are involved in the destruction of the bacterial cell wall, and in the production of ROS by the production of chitin oligomers [155]. Chitinase induction has been reported against bacterial (mainly Pseudomonas) and fungal pathogens in *Actinidia deliciosa* and *Brassica napus* subsp. *Napus*, respectively [153,156]. Additionally, leaf apoplastic chitinases and 1, 3-β-glucanases increased in response to *Cladosporium fulvum* and *Septoria tritici* diseases [157,158]. Moreover, Han et al. [159] described the apoplastic Cys-rich repeat protein 1 (CRR1), a protector of chitinase, helping the plant resistance to the pathogen releasing VdSSEP1 protease.

On the other hand, thaumatin-like proteins (TLPs) are classified as the PR-5 protein family [39]. Several studies have shown that plant TLPs are secreted in the apoplast and are involved in plant protection against biotic and abiotic stresses [160,161,162,163]. Furthermore, various studies have demonstrated that TLPs exhibit antifungal activity. For example, Shatters Jr. et al. [164] revealed that PR-5 proteins can affect fungi, either by disrupting its membranes or by hydrolyzing β-1,3-glucans of the cell walls. Additionally, Wang et al. [163] have shown that the expression of *TLP* genes in plants infected by fungi and bacteria is significantly increased.

Germin–like proteins (GLPs) are a large gene family that belongs to the cupin superfamily [165]. GLPs are involved not only in plant development but also in plant defense against biotic and abiotic stresses, including bacteria [166], fungi [167,168], salt pressures [169], and drought stresses [170]. Moreover, ADP–glucose pyrophosphatase or phosphodiesterase (AGPPas), oxalate oxidase (OxO), and superoxide dismutase (SOD) are three enzymes which are associated with GLPs [171]. In general, the primary reactions detectable in plant–pathogen recognition are the opening of specific ion channels and the formation of reactive oxygen intermediates [172]. It seems that SODs in association with GLPs protect the plant from the effect of oxidative stress by quickly converting O_2_^−^ and H_2_O into H_2_O_2_ and O_2_ [173,174]. Furthermore, GLPs also seem to be related to phytohormones, since the transcript levels of some GLPs are enhanced after application of phytohormones, including SA and ABA [175]. Additionally, a GLP with serine protease inhibitory activity (GLP-serine protease inhibitor) has been detected in the wheat apoplast and suggested to be part of a defense system against insect and bacterial proteases [157]. Moreover, GLPs are also expressed in abiotic stress. For example, expression of GLPs in tomato and *Nicotiana tabacum* plants in response to salt stress has been reported by Amini et al. [176], and Dani et al. [169], respectively.

The regulation of secreted proteases and protease inhibitors (PIs) plays an important role in apoplastic immunity [177]. It seems that proteases increase after pathogen infection systemically and locally [178]. Specifically, subtilisin-like serine proteases or subtilases play an essential role in responses to environmental stress [179]. For example, the apoplastic PR protease (P69B) is required for the induction of tomato immunity against the pathogen *Phytophthora infestans* [180]. Moreover, it has been also described that the Arabidopsis SBT3.3 gene, encoding an extracellular subtilase homologous to the tomato P69C, also plays a role in immune priming by induction SA-responsive gene [181].

### 4.2. Apoplastic Peptides Related to Plant Defense

PR gene families PR-6, PR- 12, PR-13, and PR-14, which contain protease inhibitors, defensins, thionins, and lipid transfer proteins respectively, are the so-called antimicrobial peptides (AMPs). These AMPs are usually cysteine-rich molecules that possess potential and a broad range of antimicrobial activities [118,132]. Specifically, the peptides belonging to the PR-6 family have shown effective antimicrobial activity against fungal pathogens [182]. Apoplastic papain-like cysteine proteases (PLCP) play an important role in apoplastic immunity by releasing small peptides that serve as damage-associated molecular patterns (DAMPs) [183]. In addition, it is worth noting that PLCPs and serine hydrolases (SHs) protease families are up-regulated upon pathogen infection or targeted by pathogen-derived inhibitors [184]. For example, Ziemann et al. [185] pointed out that Zip1 peptide, which is released in the presence of PLCP, leads to SA accumulation in maize leaves. Moreover, Schulze Hüynck et al. [186] identified a specific PLCP named CP1C in maize root. Surprisingly, CP1C which shares sequence homology to the Arabidopsis RD21 subfamily did not show higher activity after SA treatment of maize roots. Moreover, RCR3 is an apoplastic PLCP that acts as PR protein against fungal pathogens and nematodes [187,188].

Plant defensins and thionins are small, cysteine-rich peptides (around 5 kDa) which are grouped into plant-specific antimicrobial peptides [189,190]. There is strong evidence that some defensins are also induced upon pathogen attack, environmental stimuli, and jasmonate treatment [130,191]. For example, the defensin gene *MtDef4.2* of *Medicago truncatula* plants provides strong resistance to the fungus *Puccinia triticina* in transgenic wheat [192]. Additionally, the *Arabidopsis thaliana* apoplastic defensin type 1.1 (*AtPdF1.1*) acts to mediate defense against *Pectobacterium carotovorum* subsp. *Carotovorum* by activation of ethylene pathway in result of iron-deficiency [193]. Moreover, an increasing level of Tad1 which is a plant defensin-like has been detected in wheat in response to pathogen attack and cold temperatures. Interestingly, the *Tad1* expression is an independent defense signal and it is not regulated by the methyl jasmonate acid pathway [194].

Lipid transfer proteins (LTPs) are small, cationic, cysteine-rich peptides, which are recognized based on their role and ability to transport lipids between cell membranes. They usually have below 10 kDa molecular weights [195,196]. Extracellular LTPs have been found in various plant species, such as tobacco, cowpea (*Vigna unguiculata*), *Medicago,* and sunflower [197,198,199]. Researchers have been interested in LTPs for three main reasons: for their ability of transferring and binding to lipids, because they are one of the components of plant innate immunity, and for their clinical features [200]. Apoplastic LTPs were shown to be involved as major players in cutin formation [201]. However, much evidence showed that LTPs play a key role in plant defense mechanisms [202,203]. For example, Dani et al. [169] detected two LTPs in the leaf apoplasts of tobacco in response to salt stress. It seems that the induction of LTPs during this, stress causes deposition of cuticular material decreasing, and that way, leaf transpiration. Moreover, defective-mutants in the induced resistance gene *DIR1*, which encodes apoplastic LTPs, can still promote the SA-mediated pathway, but they have disabled the induction of systemic acquired resistance (SAR), which is one of the resistance mechanisms in plants [203,204].

### 4.3. Microbe-Associated Molecular Patterns of Proteic Nature

The recognition of microbe-associated molecular patterns (MAMPs) triggers several cellular response processes, such as alteration of ionic current between cell membranes which alkalizes intercellular space, increasing the cytoplasmic calcium ion concentration [184], and the biosynthesis of ethylene stress hormone which is activated by 1-aminocyclopropane-1-carboxylic acid-synthase (ACC-synthase). Other primary responses include activation of mitogen-activated protein kinases (MAPKs), which in turn activate transcription factors [205]. This is followed by transcription of protein-coding genes such as defensins and other antimicrobial metabolites [206].

Demonstrating the action of bacterial flagellin (a type of protein subunit of the bacterial flagellum) in the plant was the turning point in understanding innate immunity [207]. Previous studies have shown that flagellin, or more precisely flg22, inhibits the growth of Arabidopsis and leads to callose deposition in leaf tissue [208]. Flg22 led to alkalization of the tobacco cell culture suspension, and stimulated the production of ROS in the tobacco leaves. The receptors responsible for flagellin perception by Arabidopsis are the leucine-rich repeat (LRR) transmembrane receptor kinase flagellin sensitive 2 (FLS2) and the co-receptor brassinosteroid insensitive 1-associated kinase 1 (BAK1) [209,210]. It was determined that the message sent by FLS2 was through a cascade of MAPK, including AtMPK3 and AtMPK6, and led to an induction of the expression of *WRKY22* and *WRKY29* genes [205]. Moreover, when plants were treated with flg22 it protected them against a subsequent pathogen challenge, providing direct evidence that it drives an effective immune response in the plant. In addition, it seems that the flagellin perception on leaf surface may be important in stimulating the active defense response because the *Arabidopsis fls2* mutants, which had lost the flagellin receptor, were more susceptible to *Pseudomonas syringae* pv DC3000 infection [211].

The elongation factor thermal unstable, abbreviated as Ef-Tu, is another widely described MAMP protein which plays an unavoidable role in protein biosynthesis, so its presence is inevitable for bacteria. The protein was isolated and identified from *E. coli*, which had been shown to have severe MAMP activity even by disrupting its flagellin gene [212]. The receptor of this protein, called elongation factor receptor (EFR), like FLS2 also belongs to the LRR family [213].

Moreover, many plant species of the Solanaceae family detect the highly conserved nucleic acid binding motif RNP-1 of bacterial cold-shock proteins (CSPs), represented by the peptide csp22, as a MAMP. The recipient of this MAMP, which is a kind of plant receptor-like kinase (RLK), was identified in tomato and named CORE. Transfer and expression of cold shock protein receptor to *Arabidopsis thaliana* can increase plant resistance to *P. syringae* pv. tomato [214].

Unlike flagellin, EF-Tu, and CSP, the fungal protein ethylene-inducing xylanase (EIX) activity cannot be attributed to a peptide. Fungal xylanase—by releasing cell wall fragment, and therefore, the production of DAMPs—can activate various defense responses such as alkalization of the extracellular space, ethylene biosynthesis, and the production of ROS in tomato and tobacco [215,216,217]. EIX is recognized by the tomato leucine-rich repeat receptor-like proteins with a signal for receptor-mediated endocytosis, LeEIX1, and LeEIX2, of which only the latter mediates a hypersensitive response [218].

Besides proteic MAMPs, structural components of bacterial cell wall such as lipopolysaccharides (LPS) and peptidoglycan (PGN) can also act as MAMPs in the plant [219,220,221].

## 5. Hormones Found in the Apoplast and Their Functions

As mentioned above in the introduction, the apoplast consists of the intercellular space, the cell walls, and xylem. However, this space is not homogeneous throughout the plant, since a clear difference in its composition can be observed depending on the organ where it is localized and within each organ this composition varies according to the characteristics of the tissue and its function in the plant.

The hormonal levels in the apoplast are significantly lower than inside the cell and depend on the presence of transmembrane efflux carriers [222]. The main question is whether these molecules perform important functions in the apoplast. Already in 1998, Sakurai [201] mentioned the presence of auxins and cytokines in the apoplast that seemed to be involved in the transport pathway. Moreover, the concentration of these hormones was found to vary according to the different parts of the plant where the apoplast was localized and also according to the different stages of development of the plant that helped to postulate their role as growth regulators. Kramer [222] who analyzed the movement of auxins, ABA, and gibberellins in the apoplast, highlights the fact that these hormones are usually removed from the apoplast and concentrated in the cytoplasm of plant cells, playing, that way, an important role in the intracellular communication. He also showed that when reentering the extracellular, space these hormones can travel only a limited distance before reentering a cell. Although according to this author, the presence of these hormones in the apoplast does not seem to have a specific function, the auxin role in controlling shoot growth through apoplastic pH change has long been demonstrated [223]. Recently Barbez et al. [224] have demonstrated the auxin role in root growth, since endogenous auxin levels could trigger apoplastic acidification and cell elongation in *A. thaliana*. In contrast, when increasing the levels of endogenous or exogenous auxin, they observed an inhibitory effect on root growth due to the induction of a transient alkalinization of the apoplast and the subsequent reduction of cellular elongation.

On the other hand, Hartung et al. [225] showed how ABA, which is considered one of the main hormones involved in plant response to stress, moves through the xylem from the root to the different parts of the shoot where it regulates water loss and leaf growth. The ABA present in the apoplast can come from two endogenous sources, on one hand from the synthesis in the root cells and on the other hand from the synthesis in the mesophyll cells. The ABA synthesized in the mesophyll cells can be loaded to the phloem, from where it will be transported to other parts of the plant. Moreover, it is known that abiotic stresses like salinity or drought can produce an increase in the levels of ABA in the apoplast and that after acting on stomatal closure, ABA is rapidly degraded. Therefore, it seems that free ABA does not persist for a long time in the apoplast. Instead, it can be found in conjugated form as ABA-glucose (ABA-GE). Related to this, Sauter et al. [226] showed that ABA-GE levels in the leaf apoplast increase 7 times under stress conditions. One of the situations in which the role of ABA in the apoplast has been studied in detail is the activation of the synthesis of hydrogen peroxide in the apoplast, demonstrating its regulatory role concerning other hormones such as salicylic acid, jasmonic acid, and ethylene [33].

The role played by the hormones SA, JA/ET, and ABA in plant defense against biotic stresses is well known. The presence of these hormones in the apoplast is of great importance for pathogen control since many of them develop the first stages of infection in the apoplast. Scalschi et al. [11] observed that, although the content of the hormones JA, SA, OPDA, and ABA was much lower in the apoplast when compared to the content observed in leaves, it increased significantly in the presence of the pathogen. Moreover, considering that SA can act directly on the bacteria and that there are bacteria such as *P. syringae*, or *R. solanacearum* that live in the apoplast, it could well be that SA acts directly on them at this level.

One of the most studied pathosystems at the apoplast level is the *Arabidopsis-Pseudomonas syringae* pv tomato. It is well known that the earliest events of *P. syringae* infection occur in the apoplast. The interaction between auxins and cytokinins in this pathosystem has been studied [227]. Both hormones can be present in the apoplast and they usually interact antagonistically at many levels. Cytokinin can activate SA pathway in the presence of the pathogen by binding to the AHK proteins (Arabidopsis histidine kinase) present in the apoplast. This induces the phosphorylation of AHP proteins (Arabidopsis histidine phosphotransfer protein), and together with it the activation of SA signaling cascade [227] which helps the plant to defend itself against the pathogen. On the other hand, it has been shown that *Pseudomonas* is able to induce auxin synthesis through small molecules called effectors that the bacterium injects into the plant cell through the type III secretion system. Besides, the bacterium can induce the transport of free auxins in the apoplast through the AUX1 system (auxin input transporter). The presence of auxins in the cytoplasm can induce the JA/ET pathway that acts as an antagonist of SA pathway, therefore plant response against the bacteria will no longer be effective [228].

Finally, the redox status of the apoplast has been shown to be fundamental for the control of hormonal responses and the signaling cascades of the different defense pathways [229]. In general, it seems that the oxidation of the apoplast deactivates the hormones present in it. SA was shown to induce ROS accumulation during pathogen responses [230]. In addition, hormones belonging to the indole family, such as auxins, can act as antioxidants [231] since their effect is greater under reduced conditions, being able to promote the generation of hydroxyl radicals (^•^OH) in the apoplast and contribute to plant growth [232].

## 6. Concluding Remarks

In this review we have collected the current knowledge about the apoplastic response against plant stresses, including the hormones, ROS production, antioxidants, and defensive proteins and peptides. Nevertheless, in order to fully clarify the role of the apoplast in the response to plant stress, it is necessary to continue the research in extracellular compartment responses to understand the perception and transduction of the signals and the modification of metabolites. In their natural environment, plants are exposed to different abiotic and biotic stresses, and have developed an array of adaptive mechanisms, which are based on a combination of signaling pathways that allow them to react to adverse conditions and survive pathogen attacks. Due to its extracellular nature, the apoplast is involved not only in the response but also in the perception and transduction of environmental signals. This space is also considered as the first layer of stress recognition and the first battlefield against pathogens. However, the information about the presence and modulation of antioxidants, hormones, and other metabolites related to plant defense is still limited, since most of the research is focused on the response of the whole tissue and does not single out the apoplastic response.

## Figures and Tables

**Table 1:** Schematic overview of the enzymatic antioxidants.

ENZYMATIC ANTIOXIDANTS				
Enzyme	Chemical Reaction	Involved in	Cellular Location	Ref.
Superoxide Dismutase (SOD)	O_2_^•−^+O_2_^•−^+2H^+^→2H_2_O_2_+O_2_	Regulation of oxidative stress. Stress resistance or tolerance mechanisms	Apoplast, cytosol, mitochondria, chloroplast, peroxisomes	[23,69]
Catalase (CAT)	H_2_O_2_→H_2_O+(1/2) O_2_	Regulation of oxidative stress. Stress resistance or tolerance mechanisms. Plant metabolism	Apoplast, cytosol, chloroplast, mitochondria, peroxisomes	[23,27,73]
Ascorbate Peroxidase (APX)	H_2_O_2_+Asc→2H_2_O+DHA	Regulation of oxidative stress. Stress resistance or tolerance mechanisms Plant growth and physiology	Apoplast, cytosol, mitochondria, peroxisomes, chloroplast	[74,75]
Monodehydroascorbate Reductase (MDHAR)	MDHA+NADPH→Asc+NADP^+^	Regulation of oxidative stress. Stress resistance or tolerance mechanisms	Apoplast, cytosol, mitochondria, chloroplast	[23,76,77]
Dehydroascorbate Reductase (DHAR)	DHA+2GSH→Asc+GSSG	Regulation of oxidative stress Stress resistance or tolerance mechanisms Plant growth and development	Apoplast, cytoplasm, mitochondria, chloroplast, peroxisomes	[23,78,79]
Glutathione Reductase (GR)	GSSG+NADPH→2GSH+NADP^+^	Regulation of oxidative stress. Stress resistance or tolerance mechanisms	Apoplast, cytoplasm, mitochondria, chloroplast	[80]

**Table 2:** Schematic overview of the non-enzymatic antioxidants.

NON-ENZYMATIC ANTIOXIDANTS			
Enzyme	Functions	Location	Ref.
Ascorbic Acid (AsA)	-Stress perception -Redox homeostasis -Regulation of oxidative stress -Improvement of plant stress tolerance	Apoplast, cytosol mitochondria, chloroplast, vacuoles, peroxisomes, nucleus	[23,81]
Glutathione (GSH)	-Protect membranes. -Prevent protein oxidative denaturation under stress conditions -Substrate for glutathione peroxidase and gluthatione S-transferase -Metal chelator	Apoplast, cytosol, chloroplast, mitochondria, vacuole, peroxisome, nucleus	[81]
Proline (Pro)	-Osmoprotectant activity -Antioxidant capacity -Metal chelator -Signalling under abiotic and biotic stresses -Plant growth and development	Apoplast, cytosol, mitochondria, chloroplast	[82,83]
Phenolic Compounds	-Antioxidant activity -Metal chelator -Protective and signalling functions against different stresses -Plant growth and development	Ubiquitous	[84]
Polyamines	-Antioxidant capacity -Plant growth and development. -Biotic and abiotic stress responses. -Osmotic adjustment ability	Ubiquitous	[85,86]

**Table 3:** Classification of pathogenesis-related proteins and peptides.

Families	Properties	References
PR-1	Antifungal	[119]
PR-2	β-1,3-glucanase	[120]
PR-3	Chitinase type I, II, IV, V, VI, VII	[121]
PR-4	Chitinase type I, II	[122]
PR-5	Thaumatin- like	[39]
PR-6	Proteinase- inhibitor	[123]
PR-7	Endoproteinase	[124]
PR-8	Chitinase type III	[125]
PR-9	Peroxidase	[126]
PR-10	Ribonuclease like	[127,128]
PR-11	Chitinase, type I	[129]
PR-12	Defensin	[130]
PR-13	Thionin	[131]
PR-14	Lipid- transfer protein	[132]
PR-15	Oxalate oxidase	[133]
PR-16	Oxalate oxidase-like	[123]
PR-17	PRp27 Unknown	[134]
